# Care Networks, Unmet Needs, and Well-Being in Older Adults: Evidence from The National Aging Trends Study (NHATS)

**DOI:** 10.1093/geroni/igaf122.2275

**Published:** 2025-12-31

**Authors:** Sophie Arnold

**Affiliations:** Boston University, Boston, Massachusetts, United States

## Abstract

A growing body of research suggests the potential benefits of receiving help from multiple caregivers, yet little is known about whether more extensive support networks reduce care gaps and improve well-being among people aged 65 and older. Using cross-sectional data from Round 13 National Health and Aging Trends Study (NHATS), this study investigates whether having more than one primary caregiver, including both informal and formal supports, is associated with fewer unmet needs and better psychosocial outcomes. The analytic sample comprises community- and facility-dwelling participants aged 65 and older who reported needing assistance with at least one activity of daily living (ADL) or instrumental activity of daily living (IADL). Estimations include the prevalence of having one versus multiple caregivers and compare rates of self-reported ADL/IADL unmet needs and indicators of psychosocial well-being (self-reported health, feelings of pleasure, loneliness, depression, anxiety, and worry). Multivariable logistic regression models will be used to adjust for sociodemographic and health covariates to examine whether care network size independently predicts these outcomes. I hypothesize that older adults with multiple caregivers report lower levels of unmet needs, with variations in psychosocial well-being among care recipients and care provider relationship types—such as family, paid aides, friends, or unpaid care providers. These findings contribute to understanding how caregiver arrangements shape care sufficiency and psychosocial outcomes, with implications for optimizing caregiver coordination and resource allocation. Furthermore, addressing gaps in knowledge regarding multiple caregivers and older adult well-being to inform policies that improve care quality and accessibility in aging populations.

